# In Vivo Efficacy of HD0471953: A Novel GPR119 Agonist for the Treatment of Type 2 Diabetes Mellitus

**DOI:** 10.1155/2013/269569

**Published:** 2013-12-12

**Authors:** So Ra Kim, Dae-Hoon Kim, Soo Hyun Park, Young Seok Kim, Chun Hwa Kim, Tae-Young Ha, Jin Yang, Jae-Keol Rhee

**Affiliations:** ^1^Hyundai Pharmaceutical Co. Ltd., 204-4 Nonhyeon 1-dong, Gangnam-gu, Seoul 135-545, Republic of Korea; ^2^College of Veterinary Medicine, Chonnam National University, Gwangju 500-757, Seoul 135-545, Republic of Korea

## Abstract

G-protein coupled receptor 119 (GPR119) has emerged as a promising new target for the treatment of type 2 diabetes mellitus. The expression of GPR119 on the pancreatic B cells and intestinal L cells provides a unique opportunity for a single drug to promote insulin and GLP-1 secretion. In this study, we identified a novel small molecule GPR119 agonist, HD0471953, from our large library of synthetic compounds based on its ability to anti-hyperglycemic effects on T2DM murine models. We have tested the acute efficacy of HD0471953 by the oral glucose tolerance test (OGTT) with normal C57BL/6J mice. Then, chronic administrations of HD0471953 were performed to evaluate the efficacy on various diabetic rodent models. Single administration of HD0471953 showed improved glycemic control with a dose-dependent manner in OGTT with normal mice, and the insulin and GLP-1 were also increased. To identify chronic efficacy, we have observed a decline of blood glucose and fasting insulin in a dose-dependent manner of 10, 20, and 50 mpk in *db/db* mice. The results suggest that HD0471953 may be a potentially promising anti-hyperglycemic agent for the treatment of patients with type 2 diabetes mellitus.

## 1. Introduction

Type 2 diabetes mellitus (T2DM) is characterized by chronic hyperglycemia as a result of defects in insulin sensitivity [1, 2]. In patients with T2DM, depletion of glucose-stimulated insulin secretion (GSIS) is a representative feature and leads to postprandial hyperglycemia, especially in the early phase, [3]. In clinical therapy for T2DM, hypoglycemic agents such as metformin, a-glycosidase inhibitors, thiazolidines (TZDs), and sulfonylurea derivatives (SUs) used available. However, these compounds have side effects including hypoglycemia, weight-gain, and cardiovascular problems [4]. GPR119 agonist is currently receiving significant attention for its therapeutic potential as an antidiabetic agent and an appropriate treatment modality for the safe amelioration of metabolic diseases. Activation of GPR119 increases glucagon-like peptide-1 (GLP-1). GLP-1 analogs increase glucose-dependent insulin secretion through the gastrointestinal mechanism, which GLP-1 release in pancreatic b cell and enteroendocrine L cell activated GPR119 [5]. Mimicking these ligands induces GLP-1 secretion and glucose tolerance in normal and diabetic mice [6]. Numerous GPR 119 agonists orally administered have been developed to date [7].

Here, we report the in vivo characterization of the novel small molecule GPR119 agonist HD0471953 that had synthesized in Hyundai Pharm. We specifically compared the effects of HD0471953 with the dipeptidyl peptidase-4 (DPP-4) inhibitor, sitagliptin, which also increases GLP-1 level in blood stream [8], on glycemic control and insulin secretion in several animal models.

## 2. Materials and Methods

### 2.1. Chemicals and Animals

HD0471953 and sitagliptin were synthesized in house at Hyundai Pharm Inc. Prior to use in assays, HD0471953 and sitagliptin were dissolved in 0.5% carboxy-methylcellulose (CMC; Sigma-Aldrich, St. Louis, MO, USA).

### 2.2. In Vitro cAMP Measurement

Increase of cytoplasmic cAMP level by test drugs was measured in the HIT-T15 cell (KCLB, Seoul, Republic of Korea) line. The cells were cultured with RPMI1640 medium (Gibco, Gaithersburg, MD, USA) containing 10% of fetal bovine serum (FBS; Gibco) and 1% of penicillin/streptomycin solution (Gibco) in 5% CO_2_, at 37°C. The cells were incubated for 10 min with test medium, that is, Hanks buffered salt solution (HBSS; Gibco) containing 5 mM of 4-(2-hydroxyethyl)-1-piperazineethanesulfonic acid (HEPES; Gibco), 0.5 mM of 3-isobutyl-1-methylxanthine (IBMX; Sigma-Aldrich), and 0.1% of bovine serum albumin (BSA; Sigma-Aldrich), followed by cells seeded with 5 × 10^4^ cells/well of 96-well plate before day 2, in 5% CO_2_, at 37°C. Then, test drugs in test medium were treated with 10, 2, 0.4, 0.08, 0.016, and 0.0032 *μ*M to the cells for 90 min, respectively. Measurement of cytoplasmic cAMP levels afte1r drugs treatment was performed with homogeneous time-resolved fluorescence (HTRF; CISBIO, Shanghai, China). All the experiments were performed in duplicate.

### 2.3. Animals

All procedures involving animals were approved by the Animal Ethical Committee of Hyundai Pharma Inc. All animals were housed singly under a 12 hr/12 hr light/dark cycle in free feeding condition, temperature and humidity controlled rooms. Normal C56BL/6J male mice and db/db mice were obtained from NARA BIO Inc. (Seoul, Republic of Korea) and Korea Research Institute of Bioscience and Biotechnology (KIRBB; Ochang, Republic of Korea), respectively.

### 2.4. In Vivo Experiments

#### 2.4.1. Oral Glucose Tolerance Test (OGTT), Insulin, and GLP-1 Measurement

Acute pharmacological efficacy of test drugs was tested by oral glucose tolerance test (OGTT). Eight-week-old male C57BL/6 mice were fasted overnight and then orally administered 0.5% CMC (vehicle) or 20, 100 mg/kg HD0471953 through 10 mL/kg. After 30 min, glucose was given orally at a dose of 2 g/kg/10 mL, and blood samples collected. Blood glucose levels were then monitored by tail snipping at −30, 0, 20, 40, 60, and 120 minutes later glucose load. Plasma glucose and insulin levels were immediately determined using Accu-Chek Active Blood Glucose Meter (Roche, Switzerland).

To measure improvement of insulin and active GLP-1 secretion abilities by test drugs, separate experiments were performed with overnight-fasted mice. Following the administration of test drugs after 30 min, glucose bolus was loaded and the plasma samples was obtained at 20 min. The isolated plasma were analyzed with insulin and active GLP-1 enzyme-linked immunosorbent assays (ELISA; Millipore, MO, USA).

#### 2.4.2. Efficacy of Repeated Administration

The studies of chronic efficacy were performed with *db/db* mice. All animals were given vehicle or the test drugs bid administration and sitagliptin qd administration.

The vehicle groups were administered 0.5% CMC (vehicle, *n* = 7) and the test groups were administered 10, 20, and 50 mg/kg HD0471953 (*n* = 7) through 10 mL/kg. During the treatment period, the casual glucose level and body weight of each animal were checked. At 4 weeks after beginning the administration, measurement of overnight-fasting insulin level in plasma and OGTT were performed with the administrated mice. Blood was sampled from the abdominal artery and fat fad weight was immediately removed and measured. Blood samples were centrifuged at 2000 g for 10 min to obtain serum and stored at −20°C. Cholesterol and lipoprotein profiles were analyzed by an autoanalyzer (Roche Cobas, Switzerland).

### 2.5. Statistical Analysis

Values are expressed as the mean standard deviation (SD). Student's *t*-test was used to compare two groups, and multiple group comparisons were carried out using one-way ANOVA with a Dunnett's multiple comparison test. Statistical analyses were conducted using “GraphPad Prism 5” software (Graphpad Co., La Jolla, CA, USA), and a *P* value of <0.05 was considered to be statistically significant.

## 3. Results

### 3.1. Increase of cAMP Level by GPR119 Agonist

**Figure 1:**
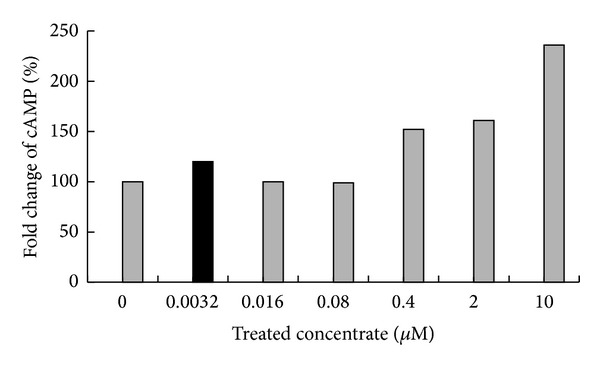
Increase in cytoplasmic cAMP level by HD0471953 in the HIT-T15 cell line. Test media containing HD0471953 were added to the cells for 90 min. Intracellular cAMP levels were measured after drug treatment by homogeneous time-resolved fluorescence.

The signaling of GPR119 was known to be triggered by elevating cAMP synthesis [9]. Thus, we have tested whether HD0471953 elevates intracellular cAMP level on the pancreatic beta cell line of hamster, HIT-T15 cells. In this test, HD0470471953 showed a dose-dependent increasing manner of intracellular cAMP levels (Figure 1). The intracellular cAMP level was around 1.5-fold from negative control at 0.4 nM and showed 2.5-fold at 10 nM of treatment HD0471953.

### 3.2. Effect of HD0471953 on Glucose Tolerance after a Single Treatment in Normal Mice

We evaluated the role of HD0471953 in the acute regulation of insulin secretion in vivo by examining blood glucose, plasma insulin levels, and GLP-1 secretion during the OGTT in normal mice.

**Figure 2:**
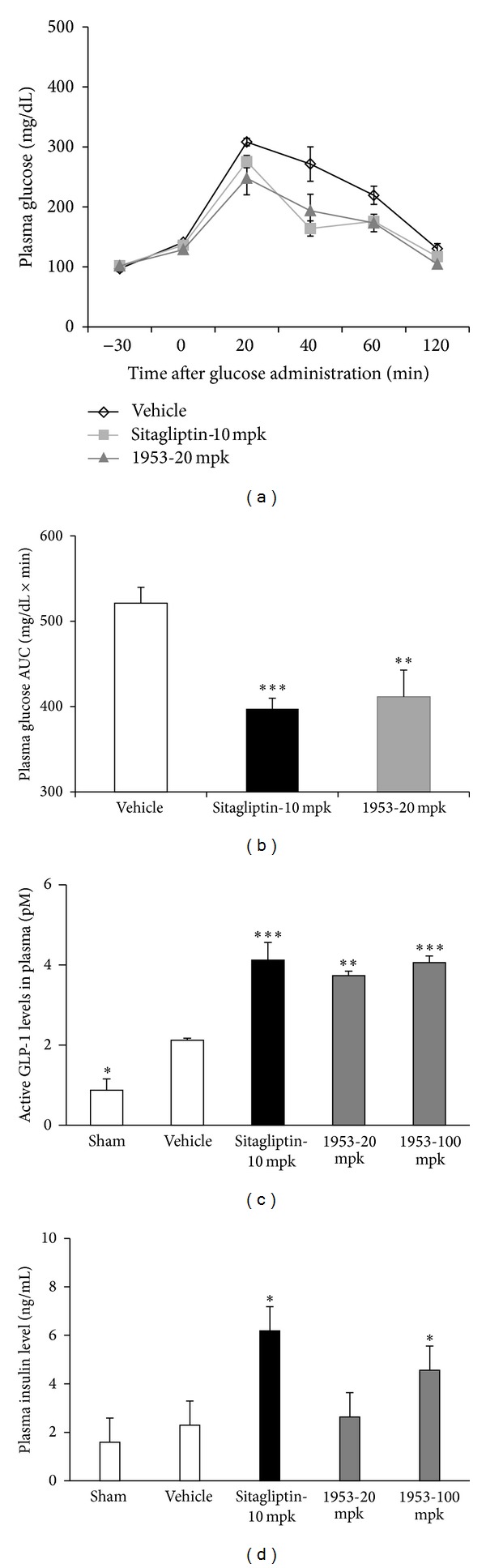
Effect of a single treatment of HD0471953 on oral glucose tolerance under normal and fasting conditions in normal mice. (a) The effect on plasma glucose levels in normal mice during an OGTT. (b) The area under the plasma glucose concentration time curve for 2 h (AUC0-2 h) in an OGTT. (c) The effect on plasma GLP-1 levels in normal mice during an OGTT. (d) The effect on plasma insulin levels in normal mice during an OGTT. Data are presented as the mean ± SE for each group (*n* = 6). **P < 0.01 versus vehicle, *P < 0.05 versus vehicle.

We also tested improved glycemic control through OGTT with single administration of drugs. Sitagliptin improved glycemic control of normal mice, administration of HD0471953 with 20 mg/kg improved glycemic control, and the glucose levels at 20 min after glucose loading was decreased (Figure 2(a)). The area under the curve (AUC) of the OGTT with single administration showed significant decrease with a dose-dependent manner after HD0471953 administration, compared to vehicle group (Figure 2(b)).

We also tested whether elevating cAMP level by HD0471953 increases insulin or GLP-1 secretion. Overnight-fasted normal C57BL/6J mice were administrated 20, 100 mg/kg dose (at “a” in Figure 2(c)) of HD0471953 before 30 min of glucose loading (at “b” in Figure 2(c)) and then after 20 min of glucose loading to analyze insulin and GLP-1 levels by ELISA. Sitagliptin, DPP-4 inhibitor, showed increase of plasma GLP-1 level (Figure 2(c), *P* < 0.001 versus vehicle group), and increased GLP-1 level may result in improved glucose-stimulated insulin secretion (Figure 2(d), *P* < 0.01 versus vehicle group). At the dose of 100 mg/kg of HD0471953, plasma GLP-1 level was significantly increased (Figure 2(d), *P* < 0.05 versus vehicle group); moreover, plasma insulin level was also significantly increased at 20 and 100 mg/kg dose (Figure 2(c), *P* < 0.05 versus vehicle group).

### 3.3. Effect of Chronic Treatment with HD0471953 in Diabetic *db/db* Mice

**Figure 3:**
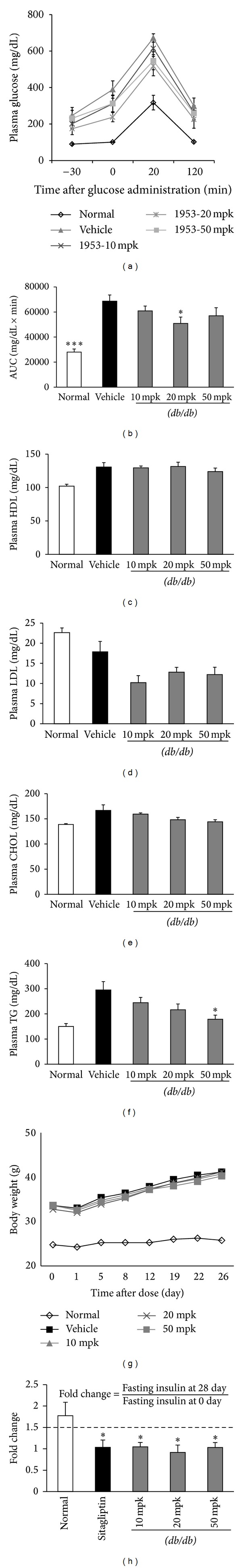
Effect of chronic treatment within diabetic *db/db* mice. A 10 mg/kg dose of HD0471953 or vehicle was administered twice daily to diabetic *db/db* mice (*n* = 7) for 4 weeks. (a) The effect on plasma glucose levels in *db/db* mice during an OGTT. (b) The area under the plasma glucose concentration time curve for 2 h (AUC0-2 h) in an OGTT. (c) Plasma HDL, (d) plasma LDL, (e) plasma CHOL, (f) plasma triglyceride (TG), (g) initial body weight and final body weight, and (h) fasting insulin level. Data are presented as the mean ± SE. *P < 0.05 versus vehicle group.

To evaluate the effects of chronic treatment with HD0471953, 6-week-old diabetic *db/db* mice were treated twice daily with 10, 20, and 50 mg/kg HD0471953 or vehicle for 4 weeks. After the treatment period, the plasma glucose fasting insulin level, initial body weight, and final body weight were checked. After autopsy, plasma high density lipoprotein (HDL) low density lipoprotein (LDL) cholesterol, plasma triglyceride (TG), epididymal fat, and inguinal fat weighed. The administration with HD0471953 decreased fasting glucose level in a dose-dependent manner (Figure 3(a)). The oral glucose tolerance test (OGTT) was performed after the drug administration during 4 weeks, HD0471953 treated groups showed lower blood glucose levels than the vehicle group, and AUC values of OGTT showed lower abundances in the vehicle group (Figure 3(b)). Moreover, the groups which were administered 20 and 50 mg/kg HD0471953 showed decreased body weight at 28 days after treatment (Figure 3(b)). High density lipoprotein (HDL) low density lipoprotein (LDL) cholesterol and plasma triglyceride (TG) demonstrated improvement compared to the vehicle group (Figures 3(e)–3(h)), but there is no significant. Initial body weight and final body weight have changed slightly (Figure 3(g)). Treatment with sitagliptin, a DPP-4 inhibitor, resulted in an increased plasma insulin level, suggesting improved glucose-stimulated insulin secretion. Sitagliptin or the 20 mg/kg HD0471953-treated groups showed lower insulin levels significantly (Figure 3(h)). Epididymal fat has no change, but inguinal fat decreased in a dose-dependent manner (Table 1).

## 4. Discussion

Various therapeutic strategies are available to improve glucose homeostasis in patients with type 1 or 2 diabetes. Although sulfonylurea derived antidiabetic drugs show strong blood glucose lowering effects, severe hypoglycemia is considered the main problem [10, 11]. TZD and other peroxisome proliferator-activated receptor (PPAR) activators have also been a popular therapeutic strategy for T2DM; however, these drugs have adverse effects such as weight gain, coronary heart disease, and heart attack [12]. Hence, drug discovery studies for new molecular targets are needed to overcome these hurdles.

Compared to other medications for T2DM, the advantages of incretin hormone-based therapy are the expectation of not only improved glycemic control, but also loss of body weight [13]. Chronic treatment of exendin-4, a long acting GLP-1 analogue, in a rodent obesity model results in improved insulin sensitivity and loss of body weight [14]. Unfortunately, all drugs based on incretin hormones do not result in loss of body weight. Although selective DPP-4 inhibitors have been reported to have strong effects on improving insulin sensitivity and glycemic control, some reports have stated that sitagliptin does not decrease body weight in obese subjects [15, 16]. Moreover, selective DPP-4 inhibitors have adverse effects related to the immune system such as rheumatoid arthritis and pancreatitis [17–19].

GPR119 is expressed on pancreatic beta cells and intestinal L cells. Its mechanism of action begins by increasing intracellular cAMP levels, and this pathway cascades to insulin or GLP-1 secretion by beta or L cells [20, 21]. HD0471953 treatment of the HIT-T15 beta cell line resulted in elevated cAMP levels in a dose-dependent manner. Acute administration of HD0471953 also increased GLP-1 and insulin secretion in normal C57BL/6J mice, which is related to improved insulin sensitivity and glycemic control in T2DM animal models [17]. These data suggest that HD0471953 may activate GPR119, which regulates insulin and GLP-1 secretion in normal mice.

Chronic administration of HD0471953 to *db/db* mice, which is a recombinant gene-induced obese T2DM model, resulted in improved glycemic control as long as the treatment lasted, suggesting that HD0471953 may improve insulin sensitivity and glycemic control. Moreover, mice treated with HD0471953 showed slight losses of body weight, although DPP-4 inhibitors have not shown this effect on body weight in several studies [15, 16]. Repeated administration of HD0471953 at 28 days resulted in lower glucose levels compared to those in vehicle treated mice. Glucose tolerance on the OGTT improved in the HD0471953 treated group at 4 weeks after administration compared to that in the vehicle-treated group. These data suggest that HD0471953 improves insulin sensitivity and glycemic control in *db/db* mice. GLP-1 agonists produce a weight loss and the DPP-4 inhibitors, conversely, appear to have a weight neutral effect [22]. GLP-1 mechanism-based glucose lowering effect shows lipid profile of HD0471953, GPR 119 agonist. HDL LDL cholesterol and TG demonstrated improvement compared to the vehicle group, but slightly. Epididymal fat level demonstrates a slight change, but inguinal fat decreases in a dose-dependent manner. This effect is minimal but still has potential.

In this study, obesity induced T2DM mice that received chronic administration of our novel GPR119 agonist, HD0471953, showed improved insulin sensitivity and glycemic control without severe hypoglycemia. Moreover, lower body weights were observed compared to those in the negative control mice. In conclusion, we have discovered and identified a novel GPR119 agonist, HD0471953, and further studies will delineate the biological functions of GPR119 and the possibility of a clinical trial.

## Figures and Tables

**Table 1:** The absolute and relative values on initial body weight, final body weight, inguinal fat, and epididymal fat after administration of HD0471953 for 4 weeks. The results are mean ± SD for 7 mice in each group.

	Initial body weight (g)	Final body weight (g)	Inguinal fat (g)	Inguinal fat (%)	Epididymal fat (g)	Epididymal fat (%)
Normal	24.75 ± 1.26	26.75 ± 0.96	0.29 ± 0.03	1.08 ± 0.08	0.29 ± 0.07	1.06 ± 0.25
Vehicle	33.66 ± 0.92	40.14 ± 2.67	2.97 ± 0.46	7.38 ± 0.80	1.71 ± 0.46	4.27 ± 1.11
1953—10 mpk	33.5 ± 1.6	39.57 ± 2.37	3.20 ± 0.35	8.10 ± 1.01	1.88 ± 0.58	4.70 ± 1.26
1953—20 mpk	32.75 ± 1.83	40.17 ± 1.47	2.97 ± 0.53	7.39 ± 1.22	1.96 ± 0.58	4.89 ± 1.48
1953—50 mpk	33.625 ± 1.41	39.5 ± 3.42	2.78 ± 0.14	7.06 ± 0.33	1.98 ± 0.63	5.00 ± 1.50
